# The Prognostic Value of Soluble ST2 in Adults with Pulmonary Hypertension

**DOI:** 10.3390/jcm8101517

**Published:** 2019-09-20

**Authors:** Laurie W. Geenen, Vivan J. M. Baggen, Robert M. Kauling, Thomas Koudstaal, Karin A. Boomars, Eric Boersma, Jolien W. Roos-Hesselink, Annemien E. van den Bosch

**Affiliations:** 1 Department of Cardiology, Erasmus University Medical Center, 3015 GD Rotterdam, The Netherlands; l.geenen@erasmusmc.nl (L.W.G.); v.baggen@erasmusmc.nl (V.J.M.B.); r.kauling@erasmusmc.nl (R.M.K.); h.boersma@erasmusmc.nl (E.B.); j.roos@erasmusmc.nl (J.W.R.-H.); 2 Department of Pulmonary Medicine, Erasmus University Medical Center, 3015 GD Rotterdam, The Netherlands; t.koudstaal.1@erasmusmc.nl (T.K.); K.boomars@erasmusmc.nl (K.A.B.); 3 Department of Clinical Epidemiology, Erasmus University Medical Center, 3015 GD Rotterdam, The Netherlands

**Keywords:** ST2, pulmonary hypertension, biomarkers

## Abstract

Soluble ST2 (sST2) is upregulated in response to myocardial stress and may serve as biomarker in adults with pulmonary hypertension (PH). This prospective cohort study investigated sST2 levels and its association with echocardiographic and hemodynamic measures, and adverse clinical outcomes in adults with PH of different etiologies. sST2 was measured during the diagnostic right heart catheterization for PH, in adult patients enrolled between May 2012 and October 2016. PH due to left heart failure was excluded. The association between sST2 and a primary endpoint composed of death or lung transplantation and a secondary composite endpoint including death, lung transplantation or heart failure, was investigated using Cox regression with adjustment for NT-proBNP. In total 104 patients were included (median age was 59 years, 66% woman, 51% pulmonary arterial hypertension). Median sST2 was 28 [IQR 20–46] ng/mL. Higher sST2 was associated with worse right ventricular dysfunction and higher mean pulmonary and right atrial pressures. Median follow-up was 3.3 [IQR 2.3–4.6] years. The primary and secondary endpoint occurred in 33 (31.7%) and 43 (41.3%) patients, respectively. sST2 was significantly associated with both endpoints (HR per 2-fold higher value 1.53, 95%CI 1.12–2.07, *p* = 0.007 and 1.45, 95%CI 1.10–1.90, *p* = 0.008, respectively). However, after adjustment for NT-proBNP, both associations did not reach statistical significance. In conclusions, higher sST2 levels are associated with more severe PH and right ventricular dysfunction and yields prognostic value in adults with PH, although not independently of NT-proBNP.

## 1. Introduction

The soluble form of suppression of tumorigenicity-2 (sST2) is a member of the interleukin-1 receptor family. sST2 is known for its release induced by myocardial strain and stress [1], but also for its involvement in type 2 immune responses [2]. Over the past years, sST2 has arisen as a promising biomarker in the heart failure population; sST2 has been investigated extensively in the case of left ventricular failure and has shown a strong prognostic value for mortality in chronic and acute heart failure patients [3,4]. Its potential relation with right ventricular dysfunction has been investigated to a much lesser extent. 

Right ventricular dysfunction is one of the major problems in patients with pulmonary hypertension (PH). Elevated pulmonary arterial pressures caused by an increased pulmonary vascular resistance can lead to progressive right ventricular failure over time [5], contributing to a very poor prognosis in these patients [6,7]. The etiology of PH is diverse and it is thought that various pathophysiologic pathways play a role in the development of this disease [8]. A previous study showed that the circulating ligand of sST2, interleukin-33, may play a role in the vascular remodeling of the pulmonary endothelium in idiopathic pulmonary arterial hypertension (iPAH) [9]. Moreover, levels of sST2 have been correlated with right ventricular dysfunction in pulmonary arterial hypertension (PAH) patients [10]. As sST2 may thus reflect both cardiac as well as pulmonary vascular remodeling, sST2 could be a valuable biomarker to monitor deterioration in patients with PH. 

The aim of this study was to assess sST2 levels between different PH etiologies, to investigate associations between sST2 and hemodynamics and measures of RV dysfunction, and to explore the prognostic value of sST2 for survival in adults with PH. 

## 2. Methods

### 2.1. Study Design

This is a prospective observational cohort study. We aimed to enroll all consecutive adult patients at the day of the diagnostic right heart catheterization for PH between May 2012 and October 2016 in our center. Diagnosis of PH was defined as a mean pulmonary artery pressure (mPAP) of ≥25 mmHg, measured by right heart catheterization in accordance with the European Society of Cardiology (ESC) guidelines [11]. Exclusion criteria were: unconfirmed diagnosis of PH due to incomplete diagnostic work-up, not treatment-naive, aged <18 years old or not capable of understanding and signing informed consent. Additionally, patients with PH due to left-heart disease were excluded. The study protocol conforms to the ethical guidelines of the Declaration of Helsinki and was approved by the local medical ethical committee. All patients gave written informed consent. 

Patients were classified according to the World Health Organization (WHO) classification of PH; PAH, PH due to lung disease, chronic thromboembolic pulmonary hypertension (CTEPH) and PH with unclear/multifactorial mechanisms (WHO5). Patients with a mixed clinical picture were grouped under WHO5. WHO 1 patients (PAH) were further stratified in subgroups according to the WHO classification [11,12]. More details of the study protocol have been published previously [13]. 

### 2.2. Study Procedures

During an inpatient visit, patients underwent physical examination by a cardiologist and pulmonary physician, 6-minute walking test, 12-lead electrocardiography, echocardiography, venous blood sampling, cardiac computed tomography, and right heart catheterization, all within the framework of PH screening. Patient characteristics and vital signs were collected. A Swan-Ganz catheter was used to obtain invasive hemodynamic measurements during right heart catheterization; pulmonary arterial pressures, right atrial pressures and capillary wedge pressures were measured. Fick’s principle or thermodilution was used to measure cardiac output. When indicated, a fluid challenge was performed to exclude PH due to left heart disease. During follow-up, patient management was according to discretion of the treating physician based on the ESC guideline [11]. Data were collected and stored in an online electronic case report form PAHTool (version 4.3.5947.29411, Inovoltus, Santa Maria da Feira, Portugal). 

### 2.3. Echocardiography and Cardiac Computed Tomography Analysis

Two-dimensional transthoracic echocardiography was performed with a commercially available ultrasound system (iE33, Philip Medical Systems, Best, The Netherlands). The guideline for echocardiographic cardiac chamber quantification was used for further imaging analysis [14]. The 4-chamber view and parasternal long axis view were used for analysis. We visually graded left ventricular systolic function as normal, mildly, moderately or severely impaired. Presence of pericardial effusion in one of the available views was scored as; mild (<10 mm), moderate (10–20 mm) or severe (>20 mm). 

Cardiac computed tomography was performed according to routine clinical practice. At the level of the pulmonary artery bifurcation, we measured both the central pulmonary artery diameter and the aortic diameter [15]. 

### 2.4. Definition and Assessment of Endpoints

The primary composite endpoint was defined as a composite of all-cause mortality or lung transplantation. The secondary endpoint was composed of all-cause mortality, lung transplantation or hospitalization due to heart failure requiring additional treatment with diuretics. Protocolled prospective follow-up visits to the outpatient clinic were scheduled with half yearly intervals. CTEPH patients who were eligible for balloon pulmonary angioplasty or pulmonary endarterectomy, were referred to a specialized center. Patients were not censored after the procedure. We retrieved information from the electronic patient records and the municipal personal records database. Patients were censored at the end of the follow-up period (1 January 2019) when they did not reach one of the endpoints. 

### 2.5. Biomarker Assessment

Venous blood sampling was performed during diagnostic right heart catheterization and performed for study purposes only. Blood samples were transferred to the local clinical chemistry laboratory and N-terminal pro b-type natriuretic peptide (NT-proBNP) was directly measured in fresh blood samples. The other serum samples were aliquoted and stored by −80 °C until batch analysis was performed. sST2 was measured in serum with a quantitative sandwich monoclonal enzyme-linked immunosorbent assay (Presage^®^ ST2 assay, CRITICAL DIAGNOSTICS, San Diego, The United States). sST2 is not significantly affected by sample free-thaw cycles and is reported to be stable up to 15 freeze-thaw cycles. The samples used to measure sST2 levels were exposed to a maximum of 2 freeze-thaw cycles with a median storage time of 3.5 [IQR 2.5–5.0] years. The upper-limit of detection was 170 ng/mL, sST2 measurements reaching the upper limit of detection were further diluted to extent the upper limit. 

In addition to the sST2 measurements in PH patients, sST2 was also measured in a healthy cohort in order to assess assay reproducibility and to obtain reference values [16]. This healthy cohort included self-declared healthy volunteers, recruited between January 2014 and December 2014. All volunteers underwent physical examination, electrocardiography, echocardiography and venous blood sampling on the same day. sST2 showed a good reproducibility with a coefficient of variation of 7.75% and limits of agreement of −5.59–7.61 ng/mL. This data has previously been published [17]. sST2 measurements were performed twice in each healthy study participant and once in study patients. 

### 2.6. Statistical Analysis

Continuous variables are presented as mean (SD) or median (inter quartile range (IQR), depending on the distribution of the data. sST2 and NT-proBNP were 2log transformed because of a skewed distribution. Comparison of sST2 levels across PH subgroups were performed using the Kruskal–Wallis test. Pearson or Spearman correlation coefficient was obtained to describe correlations between sST2 and baseline characteristics. Correlations were visualized using scatterplots. Patients were grouped based on the tertile distribution of sST2 and according to a normal or elevated sST2. Cumulative endpoint-free survival estimates were derived using the Kaplan–Meier estimator and survival between groups was compared with the log-rank test for trend. Univariable and multivariable Cox-proportional hazard regression was used to assess associations between sST2 and study endpoints. We adjusted for sex and age, and NT-proBNP in separate multivariable analyses. For sST2 sex specific reference values were used based on 97.5th percentile in the healthy volunteer cohort; >44.50 ng/mL for women and >55.85 ng/mL for men. Statistical analyses were performed using IBM SPSS Statistics (version 24). A 2-sided *p*-value < 0.05 was considered statistically significant.

## 3. Results

### 3.1. Baseline Characteristics

Of the 106 patients who were originally included in this cohort of adults with PH, sST2 was measured in 104 patients. The patient selection process is shown in Supplemental File 1. The median age was 59 (IQR 47–69) years, 64% were women and 54% were in New York Heart Association (NYHA) class III or IV. The cohort consisted of the following PH diagnoses: 53 (51%) PAH, 15 (14%) PH-lung disease, 21 (20%) CTEPH and 16 (15%) WHO5/multifactorial. (Table 1).

**Figure 1:**
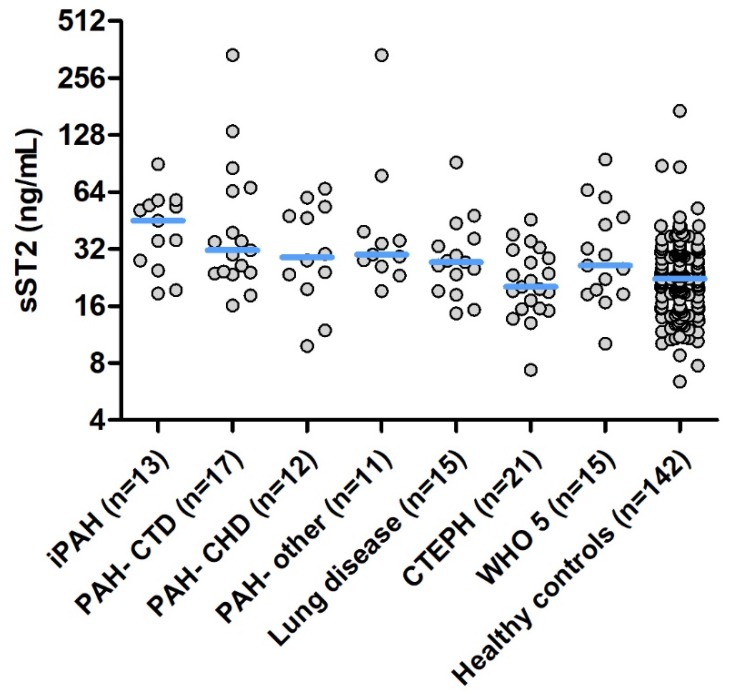
sST2 levels according to the different PH subclasses and sST2 levels in healthy volunteers. Legend: Median sST2 level in each group is indicated by the horizontal line. Y-axis is on the 2log scale. Abbreviations: iPAH = idiopathic pulmonary arterial hypertension, PAH-CTD = pulmonary arterial hypertension due to connective tissue disease, PAH-CHD = pulmonary arterial hypertension due to congenital heart disease, CTEPH = chronic thromboembolic pulmonary hypertension, WHO 5 = Pulmonary hypertension classified in group 5, PH due to multifactorial mechanisms.

Median sST2 level was 27.9 (IQR 19.6–44.9) ng/mL. sST2 levels according to the different subgroups of PH are shown in Figure 1. sST2 levels differed significantly between PH subgroups and were the lowest in CTEPH patients. An elevated level of sST2 was found in 14 women (21%) and in 7 men (18%).

### 3.2. Correlations between sST2 and Clinical Characteristics

**Figure 2:**
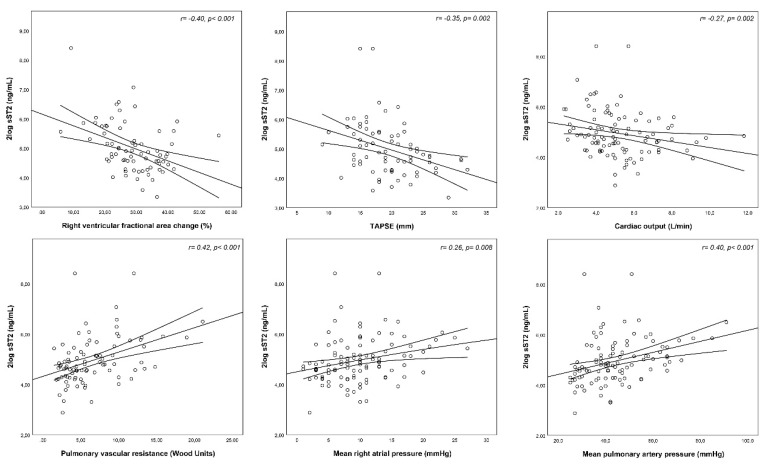
Correlations between sST2 and echocardiographic and hemodynamic measures in adults with pulmonary hypertension. Abbreviations: sST2 = soluble suppression of tumorigenicity-2, TAPSE = tricuspid annular plane systolic excursion.

Higher levels of sST2 weakly correlated with a higher NYHA class and shorter 6-minute walking distance. Both right ventricular as well as left ventricular measurements on echocardiography correlated with sST2. Regarding invasive hemodynamic measurements, sST2 showed a positive correlation with the mPAP, mean right atrial pressure and pulmonary vascular resistance. Moreover, higher sST2 was associated with lower cardiac output. (Table 2 and Figure 2) As shown in supplementary File 2, there was a moderate positive correlation between sST2 and NT-proBNP levels (*r* = 0.54, *p* = 0.001). 

### 3.3. Follow-Up

Follow-up data was complete in all patients. After a median follow-up of 3.3 (IQR 2.3–4.6) years, the primary endpoint occurred in 33 patients (31.7%) and the secondary endpoint in 43 patients (41.3%). Considering all endpoints separately, 31 patients died, 4 patients underwent a lung transplantation and 26 patients were hospitalized for heart failure. Causes of death were end-stage heart failure (*n* = 9), sudden death, presumed cardiac (*n* = 4), euthanasia in patients with end-stage pulmonary and cardiovascular disease (*n* = 3), multi organ failure (*n* = 4), kidney and/or live failure (*n* = 2), and other diverse causes (*n* = 9). Regarding the clinical management of the study patients, PH medication was initiated in 92% of the PAH patients and in 71% of the CTEPH patients. In addition, five patients (24%) with CTEPH underwent balloon pulmonary angioplasty and three patients (14%) underwent surgical pulmonary endarterectomy. 

### 3.4. Associations between ST2 and Clinical Outcomes

**Figure 3:**
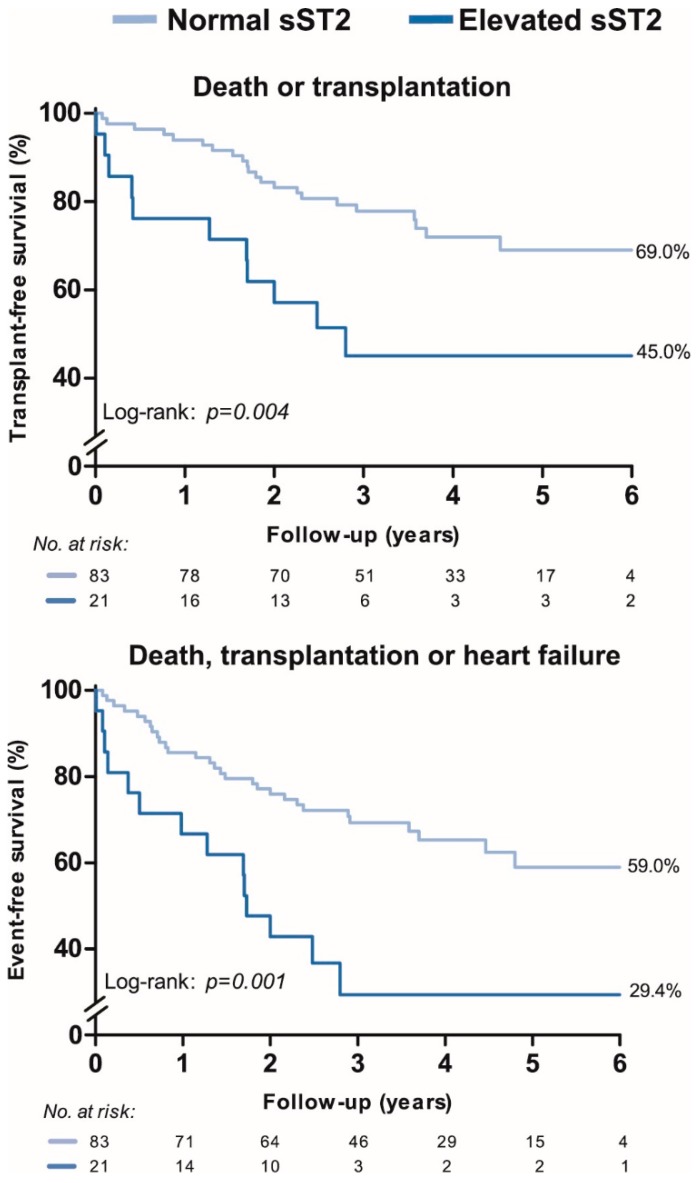
Event-free survival according to a normal sST2 or an elevated sST2 level in adults with pulmonary hypertension. Legend: Elevated sST2 was defined as 44.50 ng/mL for women and 55.85 ng/mL for men, based on the 97.5^th^ sST2 percentile in a healthy volunteer cohort. The percentage shows the cumulative end-point free survival at 6 years of follow-up. sST2 = soluble suppression of tumorigenicity-2

Figure 3 shows the event-free survival stratified according to patients with a normal and elevated sST2. Patients with an elevated sST2 were at higher risk of both the primary and secondary endpoint (*p* = 0.004 and *p* = 0.001, respectively), compared with patients with a normal sST2. Survival according to the tertile distribution of sST2, showed that patients in the first tertile (sST2 < 23.5 ng/mL) had the highest event-free survival (Supplementary file 3). Cox-regression showed a significant association between continuous levels of sST2 and the primary and secondary endpoint, independent of age and sex. However, the association between sST2 and both endpoints did not remain significant when adjusted for NT-proBNP (Table 3).

## 4. Discussion

This is the largest prospective cohort study investigating the prognostic value of sST2 in adults with PH of various etiologies. Higher sST2 was associated with a shorter 6-minute walking distance, higher NYHA functional class, right ventricular dysfunction, higher pulmonary vascular resistance and higher pulmonary- and cardiac pressures. sST2 was elevated in a substantial number of patients (20%) and these patients had a significant worse transplant-free survival. Moreover, higher sST2 was significantly associated with an increased risk for all-cause mortality, lung transplantation or heart failure; however, sST2 yielded no additive prognostic value beyond NT-proBNP.

### 4.1. Previous Studies

In 2013 Carlomagno et al. investigated sST2 levels in 25 patients with PAH of different etiologies and 10 controls and found increased levels of sST2 in PAH patients compared with healthy controls. Moreover, levels of sST2 were related to right ventricular dysfunction [10]. Median sST2 level in this study was higher than median sST2 in our study patients with PAH (43.3 ng/mL versus 34.5 ng/mL). To the extent of our knowledge, only 2 studies investigated the prognostic value of sST2 in PH patients. Zheng et al. investigated sST2 levels in 40 patients with idiopathic PAH and found a significant association between sST2 and clinical worsening. These patients were followed for a mean follow-up duration of 14 months in which 12 patients experienced clinical worsening [18]. Another study found a significant association between sST2 in 43 patients with various types of PH except due to left heart disease and found higher levels of sST2 in patients who were hospitalized for heart failure or died during follow-up [19]. This is in line with our study, which demonstrated a significant association between sST2 and the transplant-free survival in a large cohort of patients with a longer follow-up duration, whom were prospectively followed since their initial diagnosis. 

### 4.2. Pathophysiology of sST2 in PH

sST2 is part of the interleukin-33(IL-33)/ST2 ligand interaction, a cardio protective system that is upregulated in cardiomyocytes and fibroblasts as response to myocardial stress or injury. sST2 is the circulating form of ST2 and acts as decoy receptor, blocking the interleukin-33/ST2 ligand interaction [20]. Upregulation of sST2 therefore abolishes the cardio protective effects and causes maladaptive remodeling including myocardial hypertrophy, fibrosis and apoptosis [21]. 

The exact pathophysiology of PH is currently still not fully elucidated [8]; however, there are 3 processes that play a key role in the development: Vasoconstriction, vascular remodeling and micro thrombotic events [22]. Cytokines are identified to have a major contribution in the pathogenesis of PH, of which interleukins are probably the most outspoken cytokines that have been investigated in relation to PAH [23]. It has been proposed that the IL-33/ST2 ligand interaction may also be involved in the development of PAH. A previous study demonstrated that in endothelial cells from iPAH patients a marked loss of nuclear IL-33 is present and that knocking down IL-33 induced and released sST2 [9]. Hence, elevated levels of sST2 in this study may partially originate from processes associated with pulmonary vascular remodeling instead of exclusively induced by myocardial stress. This might indicate that the height of sST2 reflects both severity of pulmonary endothelial remodeling as well as progression of right ventricular dysfunction. 

In our study, sST2 was associated with hemodynamic and echocardiographic measurements, suggesting that patients with more severe PH have more ongoing myocardial and endothelial cell damage reflected by a higher release of sST2. Unfortunately, with data on patient-level we are only able to speculate about the mechanisms potentially inducing sST2 secretion. Further research is needed to elucidate the pathophysiologic involvement of sST2 in PH. 

### 4.3. sST2 and Etiologic Differences of PH

sST2 levels in all subgroups were higher compared to sST2 levels measured in the healthy volunteers, except for patients with CTEPH in whom sST2 levels seemed similar to levels in healthy volunteers. Like PAH, it has been investigated that cytokines also play a role in the pathophysiology of CTEPH [24]. An explanation for the lower sST2 levels in CTEPH patients in our study, could be presence of less severely PH, expressed by the lower pulmonary vascular resistance seen in these patients. However, the median pulmonary artery and right atrial pressure in the patients with CTEPH was equal to the median pressure in patients with PH due to lung disease. Another explanation could be the more persevering right ventricular function in these patients, as reflected by a higher fractional area change and a higher trans annular plain systolic excursion. Therefore, less sST2 may be secreted due to cardiac stress in these patients. 

### 4.4. Limitations

This study is limited by the fact that it includes patients with PH of different etiologies, introducing heterogeneity in our study cohort. sST2 levels differed between PH subgroups, therefore it is presumable that sST2 may yield a different prognostic value in each sub diagnosis of PH. The relatively small sample size restricted further subgroup analysis. Of note, patients with PH due to left heart diseases were not included in this study, this should be kept in mind when extrapolating the results to other studies. Although this is currently the largest cohort that investigated the prognostic value of sST2, it still consists of a relatively low sample size and therefore yields a limited power for multivariable statistical analyses. Blood sampling was performed in treatment naïve PH patients. However, in PAH and CTEPH patients, treatment was initiated after diagnosis of PH, during follow-up of the study. In this study we lacked the ability to adjust for treatment effect in the association between sST2 and endpoints. Therefore, the associations found in this study, reflect associations between sST2 and outcomes in adults PH patients whom were treated according to the ESC guideline [11]. Serum samples of these patients were stored by −80 °C until batch analysis took place, this could have affected serum sST2 levels. However, in our study no association was found between the storage duration and serum sST2 levels. 

### 4.5. Clinical Perspectives

According to our study, patients with an elevated sST2 level have a worse prognosis than patients with a normal sST2 level at the time of diagnosis of PH. sST2 could therefore be used for risk stratification in PH, however the independent prognostic value besides NT-proBNP seems limited, questioning the additive prognostic value sST2 might have in this perspective. sST2 has a lower biological variability than NT-proBNP [25], this could be advantageous when measuring a biomarker repeatedly over time. 

Our study suggests that high sST2 levels are seen in different PH etiologies at the time of diagnosis, except for CTEPH patients, in whom sST2 levels seemed similar to the general population. It would be interesting to investigate the influence of PH treatment on sST2 levels over time, as this may elucidate whether sST2 could be a biomarker for assessing treatment effectiveness. Serial repeated measurements of sST2 could also help to investigate secretion of sST2 in anticipation to decompensated heart failure. This study could not provide evidence whether sST2 secretion is induced by myocardial stretch, pulmonary vascular remodeling, or other potentially involved processes such as type 2 immune responses. Future studies are needed to reveal the exact mechanisms of sST2 secretion in relation to PH.

## 5. Conclusions

Levels of sST2 are higher in adults at the time of diagnosis with pulmonary hypertension compared to healthy people and differs between PH etiologies. Higher sST2 is associated with a worse exercise capacity, higher pulmonary and cardiac pressures, and with more severe right ventricular dysfunction. Moreover, sST2 is significantly associated with the risk of death or lung transplantation. Nevertheless, as sST2 yielded no independent prognostic value besides the conventional biomarker NT-proBNP, the usefulness of sST2 as prognostic biomarker in adults with pulmonary hypertension seems to be limited.

## Figures and Tables

**Table 1:** Baseline characteristics for all patients and stratified according to subgroups of pulmonary hypertension (pH).

	Complete Cases (*n*, %)	All	PAH	PH Lung Disease	CTEPH	WHO 5/mixed
		*n* = 104	*n* = 53	*n* = 15	*n* = 21	*n* = 15
**Clinical characteristics**						
Age, years	104 (100)	59 (47–69)	55 (41–66)	64 (55–72)	59 (42–73)	65 (57–70)
Sex, women n (%)	104 (100)	66 (64)	35 (66)	8 (53)	12 (57)	11 (73)
Body mass index, kg/m²	104 (100)	28.1 ± 6.6	26.3 ± 5.8	30.5 ± 6.3	31.4 ± 5.7	27.8 ± 8.5
Heart rate, beats/minute	104 (100)	80 ± 16	80 ± 16	81 ± 12	75 ± 17	85 ± 19
Systolic blood pressure, mmHg	104 (100)	127 ± 18	122 ± 15	125 ± 17	131 ± 13	133 ± 27
Oxygen saturation <90%, n (%)	104 (100)	3 (3)	2 (4)	1 (7)	0 (0)	0 (0)
NYHA class 3/4, n (%)	104 (100)	56 (54)	31 (59)	8 (53)	9 (43)	8 (53)
6-minute walking distance	89 (86)	337 ± 139	348 ± 147	309 ± 115	385 ± 130	261 ± 122
**Electrocardiogram**						
Sinus rhythm, n(%)	102 (97)	90 (87)	46 (87)	13 (87)	18 (86)	13 (87)
QRS duration, ms	100 (96)	98 (90–106)	100 (89–124)	100 (91–111)	94 (88–99)	100 (89–124)
**Echocardiogram**						
RA area, cm²	79 (76)	27.5 ± 26.2	28.0 ± 6.9	28.4 ± 8.4	24.6 ± 10.0	28.2 ± 13.1
RV end diastolic basal dimension, mm	74 (71)	51.5 ± 9.6	52.7 ± 8.1	47.8 ± 4.7	51.1 ± 12.1	50.2 ± 13.0
RV fractional area change, %	72 (69)	28.9 ± 8.6	26.7 ± 7.8	31.3 ± 6.2	33.0 ± 4.3	30.7 ± 12.7
TAPSE, mm	72 (69)	20 ± 5	19 ± 5	19 ± 3	21 ± 3	21 ± 7
LV function, n (%):	99 (94)					
Normal		65 (66)	32 (63)	10 (71)	15 (83)	8 (53)
Mildly impaired		29 (30)	17 (33)	4 (29)	3 (17)	5 (33)
Moderately/ severely impaired		4 (4)	2 (4)	0 (0)	0 (0)	2 (14)
LV end diastolic dimension, mm	80 (77)	43.2 ± 7.4	46.7 ± 5.6	46.1 ± 7.3	45.5 ± 5.6	46.8 ± 8.4
**Hemodynamics**						
mPAP, mmHg	104 (100)	42 (35–52)	46 (39–60)	37 (32–41)	37 (30–48)	42 (34–47)
mRAP, mmHg	104 (100)	10 ± 5	11 ± 6	8 ± 4	8 ± 5	11 ± 6
Capillary wedge pressure, mmHg	90 (87)	13 ± 6	11 ± 5	13 ± 4	14 ± 3	19 ± 9
Pulmonary vascular resistance, WU	86 (83)	5.5 (3.4–9.4)	7.9 (5.4–12.0)	4.4 (4.1–5.5)	3.4 (3.0–5.3)	4.1 (2.2–6.8)
Cardiac output, L/min	99 (87)	4.9 (4.0–6.2)	5.0 (3.9–5.8)	5.0 (3.9–5.8)	5.4 (4.9–6.3)	4.9 (4.0–6.8)
**Computed tomography**						
PA diameter, mm	98 (94)	34.4 ± 5.4	35.3 ± 6.0	34.9 ± 4.1	34.4 ± 5.1	31.1 ± 3.3
PA/AO ratio	98 (94)	1.12 ± 0.24	1.20 ± 0.26	1.03 ± 0.12	1.12 ± 0.24	0.98 ± 0.13
**Laboratory**						
sST2, ng/mL	104 (100)	27.9 (19.6–44.9)	34.2 (24.1–54.1)	27.3 (19.2–36.3)	20.2 (15.5–28.6)	26.2 (18.5–47.1)
NT-proBNP, pmol/L	104 (100)	60 (21–226)	120 (26–280)	60 (23–216)	24 (5–36)	31 (12–282)

**Abbreviations**: NYHA = New York Heart Association, RA = right atrial. RV = right ventricular, TAPSE = trans annular plain systolic excursion, LV = left ventricular, mPAP = mean pulmonary artery pressure, mRAP = mean right atrial pressure, PA = pulmonary artery, WU = Wood-units AO = aorta, NT-proBNP = N-terminal pro B-type natriuretic peptide, sST2 = soluble suppression of tumorigenicity-2.

**Table 2:** Correlations between sST2 and baseline characteristics.

	sST2	
Clinical Characteristics	*r*	*p*-Value
Age	−0.09	0.346
Sex	0.11	0.260
Body mass index	−0.15	0.124
Heart rate	0.28	0.005
Systolic blood pressure	−0.21	0.036
Oxygen saturation < 90%	0.08	0.417
NYHA class 3/4	0.23	0.018
6-minute walking distance	−0.29	0.007
Electrocardiogram		
Loss of sinus rhythm	0.17	0.095
QRS Duration	0.15	0.142
Echocardiogram		
Right atrial area	0.13	0.269
RV basal dimension	0.17	0.145
RV fractional area change	−0.40	<0.001
TAPSE, mm	−0.35	0.002
LV end diastolic dimension	−0.23	0.044
Hemodynamics		
mPAP	0.40	<0.001
mRAP	0.26	0.008
Capillary wedge pressure	−0.11	0.314
Pulmonary vascular resistance	0.42	<0.001
Cardiac output	−0.27	0.007
Computed tomography		
PA diameter	0.07	0.475
PA/AO ratio	0.08	0.426
Laboratory		
NT-proBNP	0.54	<0.001

Abbreviations: sST2 = soluble suppression of tumorigenicity-2, NYHA = New York Heart Association, RV = right ventricular, TAPSE = tricuspid annular plane systolic excursion, LV = left ventricular, mPAP = mean pulmonary atrial pressure, mRAP= mean right atrial pressure, PA = pulmonary artery, AO = aorta, NT-proBNP = N-terminal pro B-type natriuretic peptide.

**Table 3:** Associations between sST2 and the primary endpoint (death or lung transplantation) and secondary endpoint (death, lung transplantation or HF).

	Hazard Ratio * (95%CI)	*p*-Value
**Primary endpoint (*n* = 33)**		
sST2 (univariable)	1.53 (1.12–2.07)	0.007
*Adjusted for:*		
Age and sex	1.59 (1.17–2.16)	0.003
NT-proBNP	1.13 (0.75–1.69)	0.568
**Secondary endpoint (*n* = 43)**		
sST2 (univariable)	1.45 (1.10–1.90)	0.008
*Adjusted for:*		
Age and sex	1.50 (1.15–1.95)	0.002
NT-proBNP	1.07 (0.75–1.52)	0.712

* Hazard ratio per 2-fold higher value of sST2. Abbreviations: sST2= suppression of tumorigenicity-2, NT-proBNP= N-terminal pro B-type natriuretic peptide.

## Supplementary Materials

The following are available online at https://www.mdpi.com/2077-0383/8/10/1517/s1, Figure S1: Patient selection process of the prospective observational study including adult patients with pulmonary hypertension, Figure S2: Correlation between 2log transformed sST2 and NT-proBNP levels in adults with pulmonary hypertension, Figure S3: Event-free survival according to the tertile distribution of sST2 in adults with pulmonary hypertension.
