# Supplementary material for: The Prognostic Value of Soluble ST2 in Adults with Pulmonary Hypertension
Source: J Clin Med. 2019 Sep 20;8(10):1517. doi: 10.3390/jcm8101517 (PMC6832164; doi:10.3390/jcm8101517)
Supplement: Supplementary file 1 [file jcm-08-01517-s001.pdf]

The prognostic value of soluble ST2 in adults with pulmonary hypertension

Authors:

Laurie W. Geenen, BSc, Vivan J.M. Baggen, MD, PhD, Robert M. Kauling, MD, Thomas Koudstaal, MD, Karin A. Boomars, MD, PhD, Eric Boersma, MD, PhD, , Jolien W. Roos-Hesselink, MD, PhD, Annemien E. van den Bosch, MD, PhD,

SUPPLEMENTARY MATERIAL

List of files **Page**

Supplementary File 1. Flowchart of the patient selection process…………………………................1

Supplementary File 2. Correlation between 2log transformed sST2 and NT-proBNP levels in adults with pulmonary hypertension……………………………………………………………………..2

Supplementary File 3. Event-free survival according to the tertile distribution of sST2 levels in adults with pulmonary hypertension………………..………………………………………………………..3

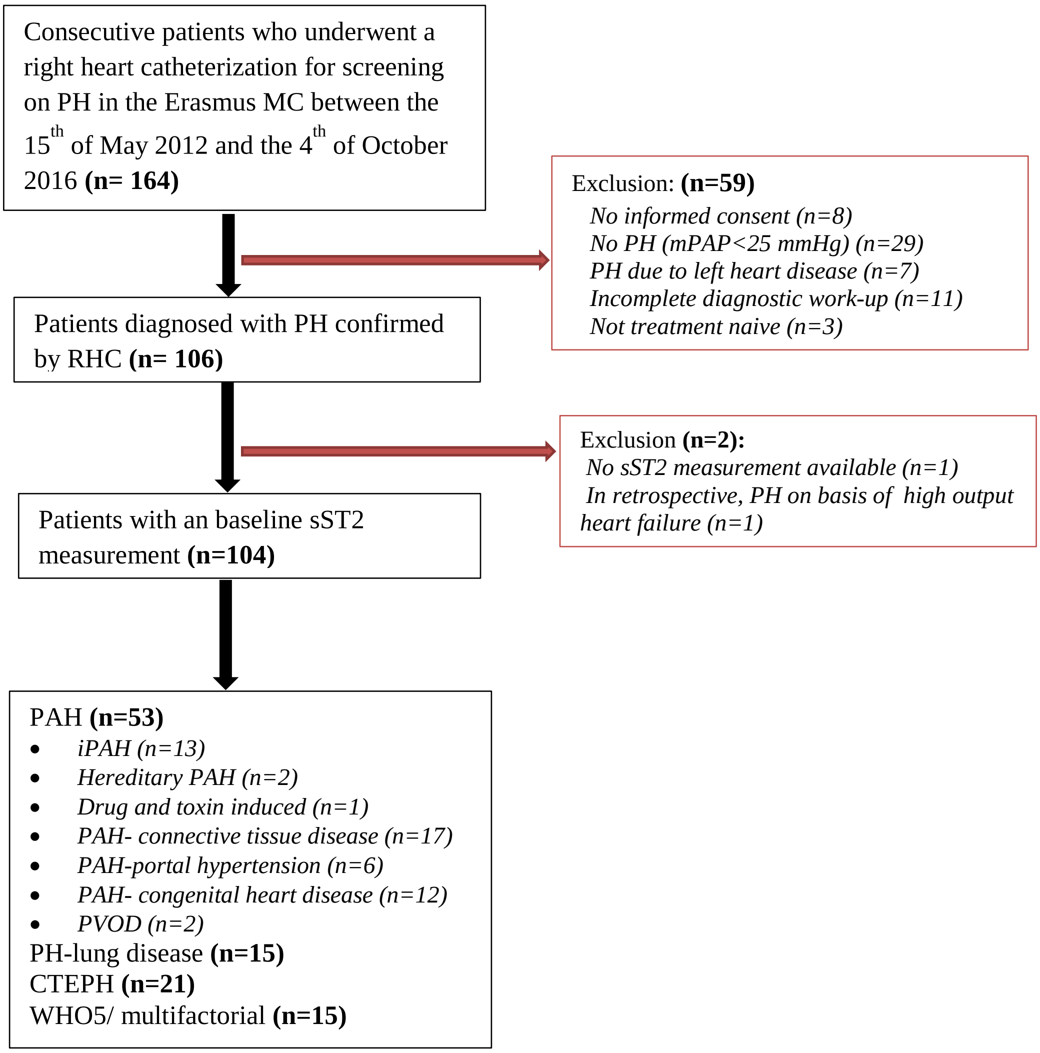

Supplementary File 1. Patient selection process of the prospective observational study including adult patients with pulmonary hypertension.

Abbreviations: PH = pulmonary hypertension, RHC= right heart catheterization, sST2= soluble suppression of tumorigenicity-2, mPAP= mean pulmonary artery pressure, PAH= pulmonary arterial hypertension, iPAH= idiopathic pulmonary arterial hypertension, PVOD= pulmonary veno-occlusive disease, CTEPH= chronic thromboembolic pulmonary hypertension, WHO5= world health organization class 5.

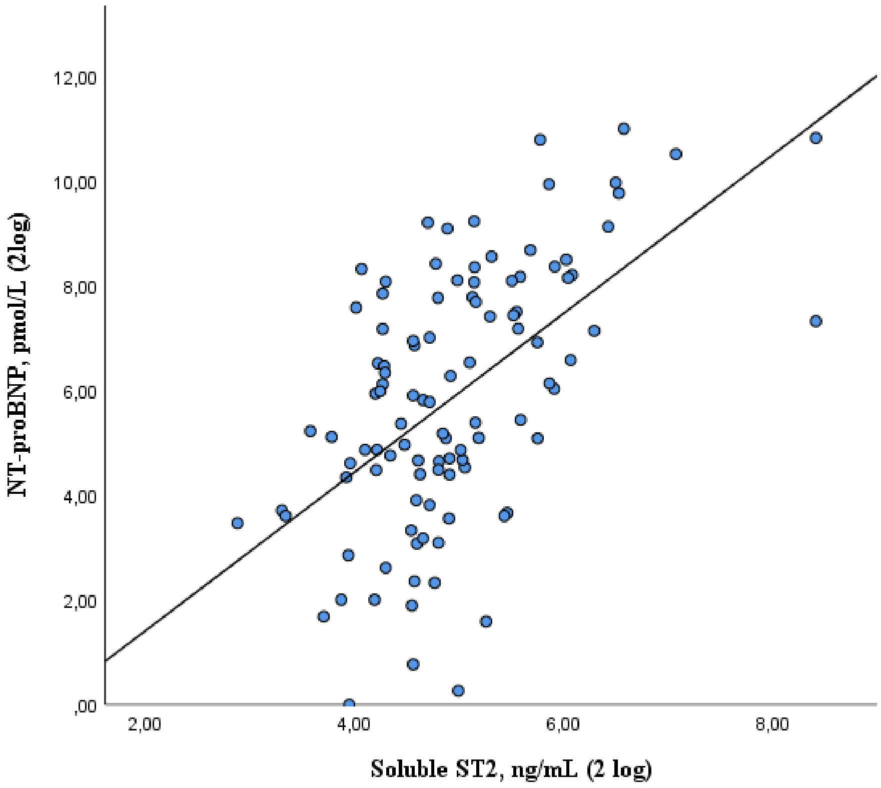

Supplementary File 2. Correlation between 2log transformed sST2 and NT-proBNP levels in adults with pulmonary hypertension.

Spearman correlation coefficient: r=0.54, p=0.001

Abbreviations: sST2= soluble suppression of tumorigenicity-2, NT-proBNP= N-terminal pro-B type natriuretic peptide.

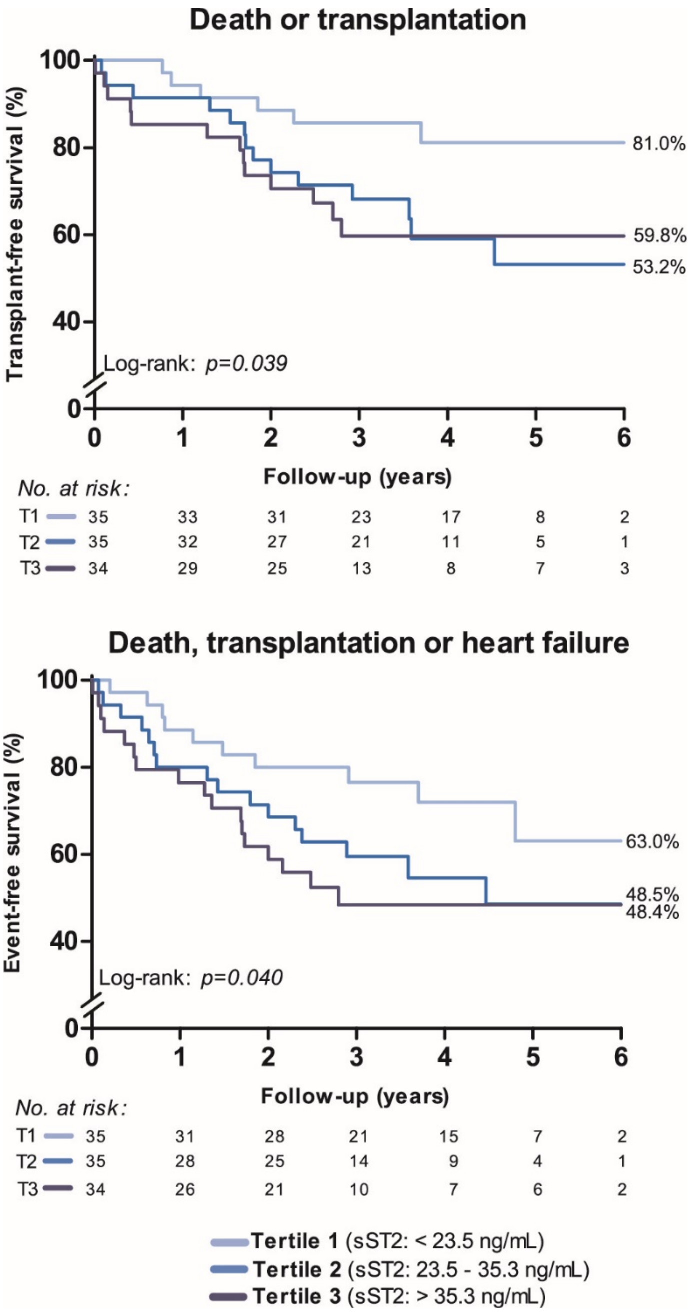

Supplementary File 3. Event-free survival according to the tertile distribution of sST2 in adults with pulmonary hypertension.

Legend: T1= first tertile, T2= second tertile, T3= third tertile. The percentage shows the cumulative end-point free survival at 6 years of follow-up according to each tertile of the sST2 distribution.
